# System Complexity in Influenza Infection and Vaccination: Effects upon Excess Winter Mortality

**DOI:** 10.3390/idr14030035

**Published:** 2022-04-21

**Authors:** Rodney P. Jones, Andriy Ponomarenko

**Affiliations:** 1 Healthcare Analysis & Forecasting, Wantage OX12 0NE, UK; 2 Department of Biophysics, Informatics and Medical Instrumentation, Odessa National Medical University, Valikhovsky Lane 2, 65082 Odessa, Ukraine; aponom@hotmail.com

**Keywords:** influenza, vaccination, pathogen interference, immune diversity, antigenic distance, winter mortality

## Abstract

Unexpected outcomes are usually associated with interventions in complex systems. Excess winter mortality (EWM) is a measure of the net effect of all competing forces operating each winter, including influenza(s) and non-influenza pathogens. In this study over 2400 data points from 97 countries are used to look at the net effect of influenza vaccination rates in the elderly aged 65+ against excess winter mortality (EWM) each year from the winter of 1980/81 through to 2019/20. The observed international net effect of influenza vaccination ranges from a 7.8% *reduction* in EWM estimated at 100% elderly vaccination for the winter of 1989/90 down to a 9.3% *increase* in EWM for the winter of 2018/19. The average was only a 0.3% reduction in EWM for a 100% vaccinated elderly population. Such outcomes do not contradict the known protective effect of influenza vaccination against influenza mortality per se—they merely indicate that multiple complex interactions lie behind the observed net effect against all-causes (including all pathogen causes) of winter mortality. This range from net benefit to net disbenefit is proposed to arise from system complexity which includes environmental conditions (weather, solar cycles), the antigenic distance between constantly emerging circulating influenza clades and the influenza vaccine makeup, vaccination timing, pathogen interference, and human immune diversity (including individual history of host-virus, host-antigen interactions and immunosenescence) all interacting to give the observed outcomes each year. We propose that a narrow focus on influenza vaccine effectiveness misses the far wider complexity of winter mortality. Influenza vaccines may need to be formulated in different ways, and perhaps administered over a shorter timeframe to avoid the unanticipated adverse net outcomes seen in around 40% of years.

## 1. The Excess Winter Mortality (EWM) Calculation

This and previous studies use a rolling/moving EWM calculation which shows deaths in the four ‘winter’ months as a percentage difference to the preceding eight non-winter months. Since winter infectious outbreaks can occur early or late, and that ‘winter’ is more objective near the equator the calculation is performed as a rolling or moving percentage difference. Hence, we start at the first 12-months data, where the EWM calculation is:

EWM = average deaths (September to December) ÷ average deaths (January to August).

Move forward one month and recalculate, etc. EWM for that winter is the maximum value. In the northern hemisphere temperate zone, the EWM most commonly reaches a maximum at the 12-months ending in March. The EWM calculation is very reliable and the only instances when it will give an answer lower than actual is when there is a highly unusual summer heat wave or when the winter of the preceding year occurs very late, and the current winter occurs early. Both can be overcome by retrospective adjustment.

## 2. Introduction

During the past 70 years both influenza epidemics and vaccination have been largely viewed from a narrow single pathogen perspective. From this point of view, efficient epidemic control for an antigenically variable pathogen, such as influenza, is achieved by regular immunization of most of the human population—within the constraints of cost benefit [1]. However, more recently it has become apparent that influenza outbreaks, influenza vaccination and the observed excess winter (all-cause) mortality operate within a complex system of:

1. human immune variability which includes gender, chronological and immune age, individual history of host-virus and host-antigen interactions, ethnicity, persistent pathogens, genetic mutations, epigenetic factors, psychological stress, and metabolic health [2,3,4,5,6,7,8],
2. the role of meteorological variables on influenza (and other respiratory pathogen) survival and transmission [9,10,11,12,13,14],
3. influenza virus evolution [15,16],
4. the variable spatiotemporal spread and distribution of influenza strains and mutations (clades) each year [17,18].
5. the pathogenicity of influenza being the result of a complex system of interactions between the influenza viruses, other viruses, the host, anthropogenic interventions, and secondary infections [19,20,21].
6. the totality of winter pathogen-induced deaths which is a composite of (co)infection by multiple pathogens [22,23,24,25].

All these factors combine to give remarkably high inter- and intra-national variation in excess winter mortality (EWM) during each influenza season, along with highly complex long-term trends [26], and equally remarkable variations in vaccine effectiveness between seasons [27]. A recent study has suggested that the long-term average for the net effect of influenza vaccination upon EWM was undetectable [26], because the whole system is far more complex than just influenza and influenza vaccination. This same observation has also been noted in two other large studies where, during a time of rapidly rising influenza vaccination in the elderly, no net reduction in EWM could be discerned [28,29].

As an example of the shift to a more complex system view of influenza epidemics and influenza vaccination, Table 1 shows the results of a search using Google Scholar regarding the number of hits for a variety of influenza-related complex system queries. Clearly some of these hits may not be relevant or be duplicates, nevertheless they indicate a general trend toward system complexity thinking.

Key features of complex systems are unexpected dynamic and unexpected outcomes of interventions, called ‘emergent behavior’, bifurcation (or tipping) points where a division into branches or sub-groups occurs, i.e., fractal behavior, and unrealized multiple equilibria or steady states [30,31,32,33,34,35,36]. The population dynamics of pathogens and pathogen-host interactions depend on multiple factors, including natural and anthropogenic factors, along with other hidden factors, and are often underestimated.

Regarding the multiple equilibria, the immune system does indeed exist in multiple steady states [37,38,39,40,41,42]. Such immune-endocrine steady states can correspond to certain illnesses, such as Gulf War illness and chronic fatigue syndrome [38]. Infectious disease models, likewise, show multiple equilibria [43,44,45]. In such a complex system, influenza vaccination may yield unexpected outcomes, i.e., may benefit one group, have no effect on another or cause disbenefit in another.

Hence, assessing overall, or net, vaccine benefits in such a complex system may be less than straightforward. One study which used a systems biology approach screened multiple morbidities, anthropometric measurements, and biochemical parameters and concluded that only relative lymphopenia (decreased percent of lymphocytes in WBC differential), OR 0.94, (95% CI 0.88–0.99); vitamin B12 deficiency OR 0.99, (0.99–1.00); and hyperhomocysteinaemia OR 1.15 (0.99–1.32) showed potential to predict an influenza vaccine response (as antibody production) for the 2003/04 trivalent vaccine [46]. For more accurate evaluation of influenza vaccine efficacy parameters of cellular immunity and their interaction with other factors should also be measured. However, this seemingly indicated that biochemical health (howsoever determined) may be a neglected key parameter in determining antibody production (but not necessarily vaccine efficacy). Hence factors such as obesity and multi-morbidity are indirect measures of their effects upon the individual’s biochemical balance.

The above issues are neatly summarized in a recent study, which demonstrated that all-cause excess winter mortality (EWM) is the output of an exceedingly complex system which exhibits long-term undulations in EWM—and therefore implies the existence of potential hidden and unexpected ‘emergent’ outcomes [26].

The methodology behind the calculation of EWM has been extensively discussed in two previous articles [26,47]. In summary, it calculates the percentage of excess winter deaths for the four winter months relative to the eight non-winter months. The calculation is performed on a running/moving basis to detect which four-month period gives the maximum difference. This then allows for years in which influenza outbreaks may occur very early or late and allows for winter in the southern hemisphere.

This study contains several parts. In the first is an overview of the international trends in EWM, especially focusing on high inter- and intra-national spatiotemporal granularity in each year and what this may imply regarding the complexity of each winter. We then investigate if there is a relationship between international EWM and proportion of those aged 65+ who receive influenza vaccinations. This is achieved using two data sets, namely, age 65+ vaccinated data and doses of influenza vaccine distributed. The latter is then converted into an age 65+ vaccinated equivalent. Both are previously described [26].

Rather than conduct this analysis over a longitudinal scale, as was done previously [26], the analysis focuses on each winter and, specifically, on the differences in EWM as a function of the differences in elderly influenza vaccination rates between world countries. The emphasis is on the detection of unexpected or emergent outcomes which complexity theory indicates should exist. EWM is a key tool, because it measures the net effects inherent in each winter and can thereby detect unexpected or emergent behavior.

## 3. Materials and Methods

### 3.1. Sources of the Data

Monthly deaths and rolling/moving EWM calculations for a range of countries were taken from a previous study [26]. Proportion of persons aged 65+ vaccinated in each country over time was also taken from the previous study [26]. Data relating to vaccine effectiveness in those aged 65+ in the USA was from the Center for Disease Control and Prevention (CDC) [11]. Annual estimates of adult obesity since the 1980s for world countries was obtained from the World Health Organization (WHO) [48] and the Global Obesity Observatory [49].

### 3.2. Adjusting EWM for Each Country to a US-Equivalent

The USA has the most available data for rates of vaccination in those aged 65+, plus EWM data [26]. It therefore makes sense to adjust the EWM of all other countries to a US-equivalent. This was achieved by adjusting the data from all countries using the median EWM for each country compared to that of the USA. EWM data for each country was adjusted such that the adjusted EWM has a median equal to that seen in the USA as detailed in the previous study [26].

### 3.3. Method for Excluding Outlying EWM Values

For smaller countries with lower deaths per annum there can occasionally be statistically high/low values for EWM. For countries lying close to 0% vaccination, adjusted values of EWM lower than 5% and higher than 20% were trimmed. For countries with higher rates of vaccination a different rule was applied, such that values were only excluded from the study if they were markedly higher/lower than all other countries. This sometimes occurs for data from smaller countries where Poisson randomness becomes more significant.

Exclusion is required to avoid the undue effect of outlying values on linear regression based on the least-squares methodology. The distance squared means that outlying values are unduly weighted in the regression. Future studies on this topic could use weighted regression without trimming; however, this is unlikely to make a material change to the conclusions.

### 3.4. Adjustment of EWM for Obesity Relative to the USA

**Figure A1:**
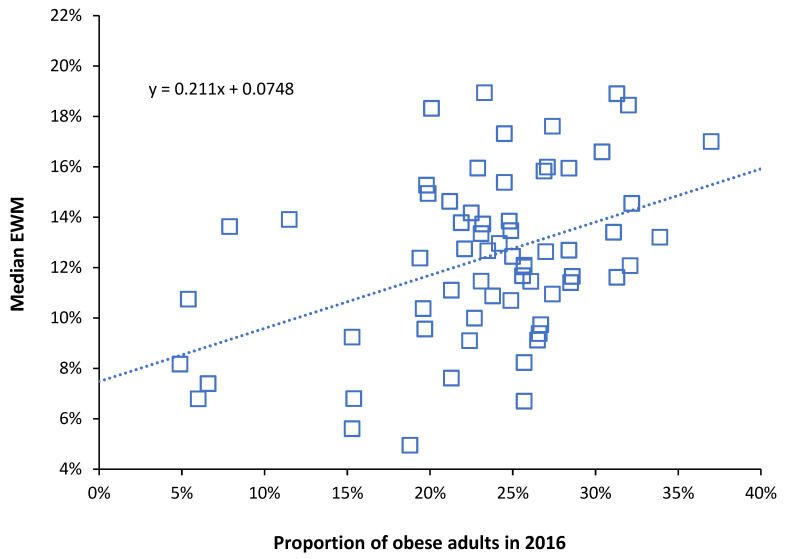
Proportion of obese adults in 2016 versus the median excess winter mortality (EWM) for 65 world countries. Footnote: Median EWM data has not been adjusted for latitude and the resulting slope is likely to be an over-estimate. In a previous study regarding the effects of elderly obesity on latitude adjusted median EWM for US states the slope of the relationship was 0.07 [26]. Hence the true slope is probably somewhere between these two values. The most likely value of the slope was determined by applying values of the slope between 0.02 and 0.3 to the study data. The R-squared value reached a maximum at a slope of 0.12 (as per Table S1 in the Supplementary Material). Note that the proportion of obese elderly aged 65+ is very similar to overall adult obesity. As this study demonstrates adjusting for the obesity differential to the USA makes no effect on the outcome.

As in the previous study EWM data for each year was adjusted to give the equivalent to that in the USA [26]. Obesity data for world countries in 2016 was plotted against the median EWM for each country over the period 1990 to 2020. This gave a slope of 0.2, i.e., for each percentage point increase in obesity the median EWM increases by 0.2% (See Figure A1 in the Appendix A).

**Figure A2:**
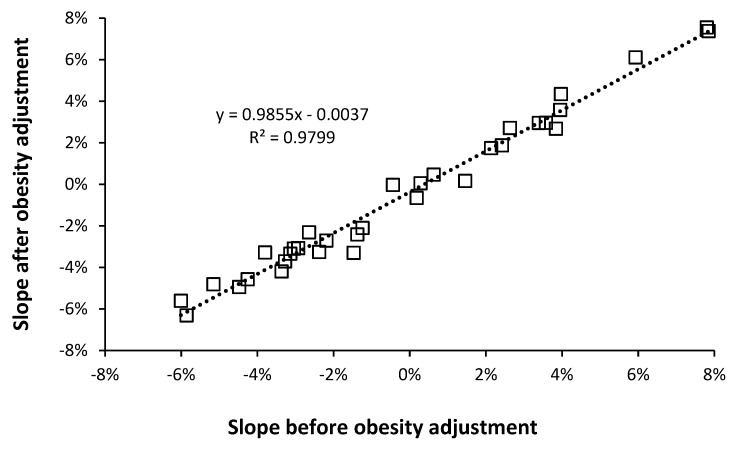
Relationship between the slope of the relationship between EWM and proportion aged 65+ vaccinated before and after obesity adjustment. Footnote: The obesity adjustment factor is a 0.12% increase in EWM for a 1% increase in adult obesity as per Table S1 in the Supplementary Material.

This was higher than that observed in an earlier study [26], and so the effect of the slope upon the relationship between EWM and influenza vaccination was evaluated for values of the slope between 0.02 and 0.3. (Table S1 in the Supplementary Material). The R-squared for this relationship reached a maximum at a value of the slope equal to 0.12 (Figure A2 in the Appendix A). Since all countries in this study had a level of adult obesity less than the USA the adjustment factor for EWM was then as follows: Obesity Adjusted EWM = Raw EWM + [adult obesity in USA (%) − adult obesity in country A (%)] × 0.12. This calculation is repeated for each year.

### 3.5. EWM in US States since 2008

Monthly deaths have been available for US states since January 2008 [26]. The median EWM for each state was calculated up to the winter of 2019/20 and adjusted EWM was calculated as per Section 3.2. The proportion of persons aged 65+ vaccinated for influenza for each state was estimated by multiplying the US average by the ratio of nursing home residents vaccinated in each state relative to the US average [50].

### 3.6. Data Manipulation

All data was manipulated using Microsoft Excel. Linear regression was performed using the “Add Trendline” function.

## 4. Results

### 4.1. EWM Shows Extreme Spatiotemporal Volatility

**Figure 1:**
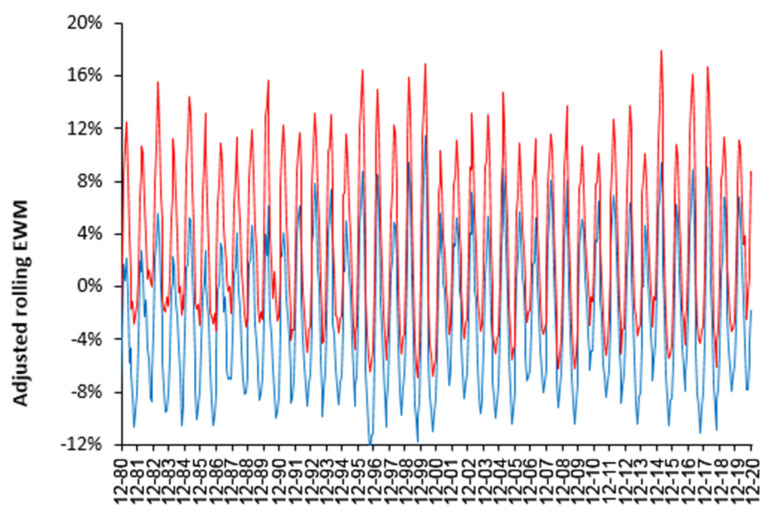
Upper and lower quartile for a rolling EWM calculation for 134 countries and 34 states/provinces (Australia, Canada, Germany). Due to data availability, there is a maximum of 143 countries/states for each winter.

Excess winter mortality (EWM) varies considerably from one year to the next and Figure 1 shows this variation using a rolling/moving EWM calculation for up to 143 countries and states/provinces. In Figure 1 the EWM for each country has been adjusted up/down by the ratio of the median EWM for the USA divided by the median EWM for each country [26]. Note that the inter-quartile range only covers the 50% of countries closest to the international median for each winter.

The variation is illustrated by showing the international upper and lower quartile. As can be seen, EWM reached a minimum in the winters of 2000/01 and 2013/14 with a median = 8.8% (for clarity the line for the median is not shown) and a maximum in the winters of 1999/00 (median = 17.2%) and 2014/15 (median = 16.5%). However, the inter quartile range (IQR) reached its maximum extent of 16.2% for the winter of 1989/90 and its minimum extent of 6.9% for the winter of 2019/20, just before the COVID-19 pandemic. A high IQR indicates extreme differences around the world. The sharpness of each winter peak measures the differences in timing between countries. Note the cluster of 3 high years between 2014/15 and 2017/18 and 4 high years between 1995/96 and 1999/00. Influenza vaccination is therefore being applied into a system showing high intrinsic international variation, clustering of high EWM, and year-to-year volatility.

**Figure 2:**
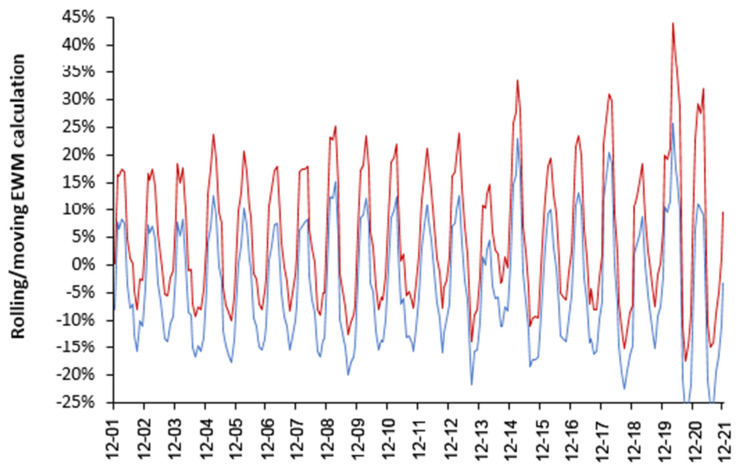
Upper and lower quartile for a rolling/moving EWM calculation covering 417 local government areas (LGA) across the UK each with fewer than 5000 deaths per annum. EWM for each LGA has not been adjusted.

To determine if a wide IQR is specific to world countries Figure 2 shows an identical rolling EWM analysis to that in Figure 1 using 417 local government areas (LGA) within the UK (2001 to 2021). Figure 2 also contains the first and second wave of COVID-19 as an illustration of an infectious outbreak with high spatiotemporal variation.

The key point is that the range for the upper and lower quartile within the UK is very close to that for world countries, even though world countries range from near the equator to close to the poles, i.e., even the within-country variation in EWM is profoundly high.

The IQR in Figure 2 is not an artefact of LGA size since the median size (as deaths per annum) in the two tails is not greatly different from the middle 50% of EWM values (the IQR) and is at least 3-times higher than the minimum size threshold (400 deaths per annum) applied to the international data. Indeed 50% of UK LGA have over 1500 deaths per annum and 75% are higher than 1000 deaths per annum.

The second point is that both the upper and lower quartile in Figure 2 is made up from unique winter behavior, which is indicative of differing spatial spread of the causative agents—as observed for the two COVID-19 waves (last two peaks). Note that the events in the winters of 2014/15 and 2017/18 have an upper quartile equal in magnitude to the second COVID-19 winter. It is proposed that it is the spatiotemporal spread of pathogens within the UK which drives the variation, of which influenza made a significant (but not exclusive) contribution prior to the arrival of COVID-19.

The winter of 2013/14 in the UK, which had the lowest EWM, was remarkably mild and wet [14], which seemingly led to low levels of influenza (and other winter pathogen) activity and mortality [15]. This is consistent with cold-dry conditions favoring influenza spread in temperate countries [13]. Hence, low EWM is associated with low levels of winter pathogens while high EWM is associated with high levels of pathogens (as per COVID-19).

While high spatiotemporal variation in infectious outbreaks is well known to epidemiologists, the implications to inherent complexity and unexpected or emergent behavior may have been largely overlooked.

### 4.2. Influenza Vaccination in the Elderly

Rates of influenza vaccination vary widely between world countries. The median for vaccination rates between countries in those aged 65+ ranges from 4% in 1988/89 (maximum 45%) to 48% in 2019/20 (maximum 85%). Countries with highest vaccination rates for age 65+ changes over time with the Netherlands highest between 2000/01 to 2008/09 (range 76% to 83%), Mexico was the highest in 2009/10 during the Swine flu pandemic (88.2%), and briefly highest between 2013/14 and 2014/15 (79% to 82%), while South Korea was highest in 2011/12 and 2012/13, and from 2015/16 onward (up to 86% vaccinated) [51]. Hence there is a sufficiently wide range in vaccination rates for every year during the study to enable evaluation of the role of vaccination on EWM.

**Figure 3:**
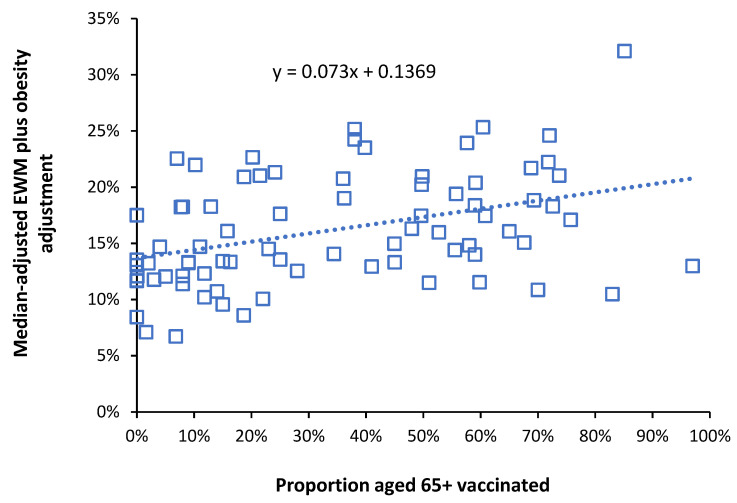
Slope for the relationship between obesity adjusted EWM and proportion aged 65+ vaccinated for 80 world countries during the winter of 2017/18. Linear regression as the dotted line.

If influenza vaccination has a net protective effect the slope of the relationship between EWM and proportion aged 65+ vaccinated should have a negative slope. Figure 3 gives one example of such analysis for the winter of 2017/18 where the resulting slope is positive (disbenefit) rather than negative. The R-squared for Figure 3 was 0.156. Such low values of R-squared are typical for each year and arise as a direct consequence of the high international variation demonstrated in Figure 1 and Figure 2.

Note that the winter of 2017/18 in both Figure 1 and Figure 2 shows unusually high EWM. A slope of 0.073 in Figure 3 implies that a population with 100% vaccinated elderly persons will have an EWM (at the US equivalent) which is 7.3% higher than if there had been no vaccination, i.e., an adverse outcome. In Figure 3 raw EWM for each country was first adjusted to the equivalent to the USA using the median EWM and then further adjusted to match US levels of obesity via the effect of obesity on international EWM (as per #4 below).

Similar analysis to Figure 3 was conducted each year from the winter of 1987/88 through to 2019/20. Four alternative scenarios for each year were performed, namely:

1. Data from all available countries
2. The 50 countries with the highest number of years of available data
3. #1 plus data from US states (available for 2007/08 onward) [26]
4. #1 plus additional adjustment of each country for difference in obesity relative to the USA [48,49]

**Figure 4:**
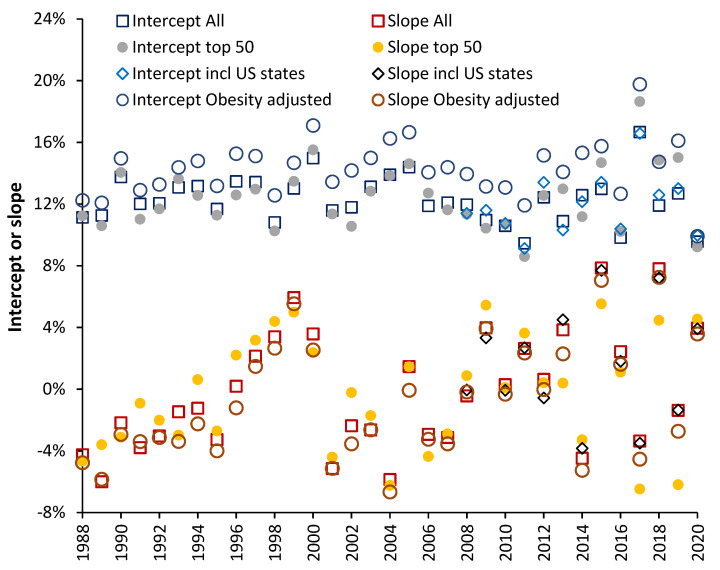
Slope and intercept of the relationship between adjusted excess winter mortality (EWM) and proportion aged 65+ vaccinated. A negative slope implies that the net effects of influenza vaccination are beneficial while a positive slope implies the opposite.

These four scenarios were performed to demonstrate that the resulting slope and intercept are robust. The resulting values for each scenario are given in Figure 4.

The year shows the winter ending in that year, hence, 1989 = 1988/89 through to 2020 = 2019/20. EWM for 2020 was calculated at the end of March to avoid distortion due to the COVID-19 pandemic. In this study complete or partial data was available for 97 countries. The minimum available data pertained to 50 countries in 1987/88 through to a maximum of 85 in 2013/14 and 2014/15. Countries were ranked by years of available data. The top 50 group was an arbitrary division. The most complete data were for members of the European Union, Australia, New Zealand, USA, and Canada. Table S2 in the Supplementary Material shows the number of available countries for all countries and the top 50 countries. The top 50 countries contain five small countries (Greenland, Malta, Iceland, Liechtenstein, and Luxembourg) where Poisson randomness leads to occasional instances of EWM values which were excluded. Hence, count of available data for the top 50 range from 37 in 1987/88 through to 50, median is 46. For the 47 other countries available data ranges from 11 to 36, with a median of 27.

From Figure 4 the intercept for the data with additional obesity adjustment is slightly higher than the other scenarios. This is because all countries have lower levels of adult obesity compared with the USA. The gap between obesity in the USA and other countries rises with time. The maximum gap was a 12.9% difference in 1980 rising to a 34.7% (percentage point) difference between Japan and the USA in 2019. Hence their EWM is adjusted upward by a maximum of between 1.5% (in 1980/81) and 4.2% in 2019/20. A higher intercept is therefore to be expected.

The slope of the relationship after obesity adjustment is highly correlated with the slope before obesity adjustment (R-squared = 0.9798) but values of the adjusted EWM are 0.37% higher than the unadjusted slope (see Figure A2 in the Appendix A) because the intercept has been increased. This is consistent with a slightly higher intercept leading to a 1.45% reduction in the slope (Table S1 in the Supplementary Material).

**Figure A3:**
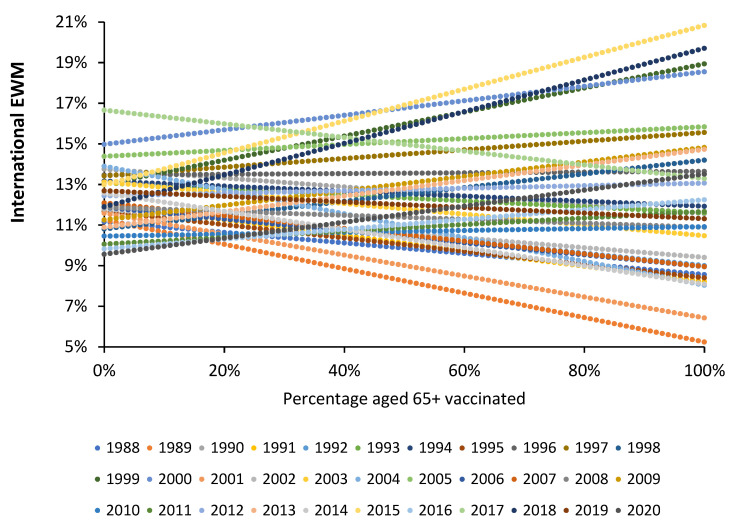
Slope and intercept of the linear regression between international EWM values and percentage elderly vaccinated over all the years included in this study. Footnote: The slope and intercept are not correlated (R-squared = 0.022). The standard deviation between the lines of best fit reaches a minimum at 0% to 15% vaccination. The intercept at 0% vaccination ranges from 16.6% in 2016/17 down to 9.6% in 2019/20. As can be seen there are only 2 years with a very high net protective effect (1988/89 and 2000/01), but four years with a very high net disbenefit (1998/99, 1999/2000, 2014/15, 2017/18) and all these years correspond with very high EWM. Hence it is difficult to claim that influenza alone was responsible for the higher deaths in these four years.

The slope for the top 50 countries shows highest divergence mainly because there are fewer data points (as discussed above) and hence the uncertainty in the slope will be higher. The scenario including US states has the highest number of data points each year. The intercept and slope for each year is also shown in Figure A3 in the Appendix A. A summary of the available data is given in Table S2 in the Supplementary Material which also includes an estimate of the standard deviation of the slope by comparing methods #1, #3 and #4 above.

As can also be seen, the slope of the relationship ranges from −6.7% for the winter of 2003/04 (a net beneficial effect) up to +7.2% for the winters of 2014/15 and 2017/18 (net disbenefit). In addition, cyclic behavior is also apparent with the first cycle rising to a maximum in the winter of 1989/99. After 1998/99 there is a trend down to 2003/04, another trend up to a plateau, and a period of instability beyond 2012/13. Roughly half the data lie above/below a slope of 0%, i.e., the point of no net effect.

### 4.3. Comparison with a Previous Study

A previous study gave an apparent zero slope for the relationship between adjusted EWM, and proportion elderly vaccinated using data over a 30-year period [26].

**Figure 5:**
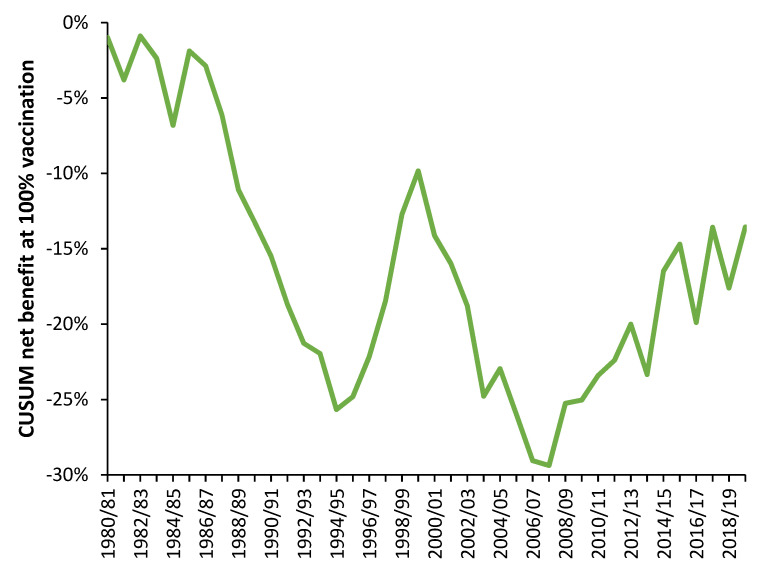
CUSUM of the annual value of the slope.

As was pointed out in the previous study [26], it would be highly unlikely for influenza vaccination to have zero net effect on EWM in every single year and to this end a cumulative sum of differences (CUSUM) is relevant. The CUSUM of the slope over time is given in Figure 5. A CUSUM is a useful tool to reveal when the behavior shows a sudden transition [52], which leads to a change in slope in the CUSUM. Over this 39-year period there are two extended periods of net benefit, namely, 1986/87 to 1994/95 and 2000/01 to 2006/07, and two periods of net dis-benefit, namely, 1995/96 to 1999/00 and from 2008/09 onwards.

There are two periods of high instability, namely 1980/81 to 1985/86 and 2013/14 to 2019/20. Over the entire 39 years the overall average net benefit is only 0.4% (percentage point) reduction in EWM per annum, at a theoretical 100% of elderly vaccinated, i.e., a CUSUM of −15% divided by 39 years. In comparison, for the 14-year period ending 1994/95 the average net benefit is a 2% (percentage point) reduction in EWM per annum at 100% elderly vaccination. The perception of the net benefit of influenza vaccination depends entirely upon when the study is conducted. Indeed, most studies only cover a limited number of years.

### 4.4. Further Validation of the Results

The previous study [26] also included a second large data set where EWM was plotted against total vaccine doses per 1000 total population (all-age) which covered the winters 1980/81 through to 2012/13.

**Figure 6:**
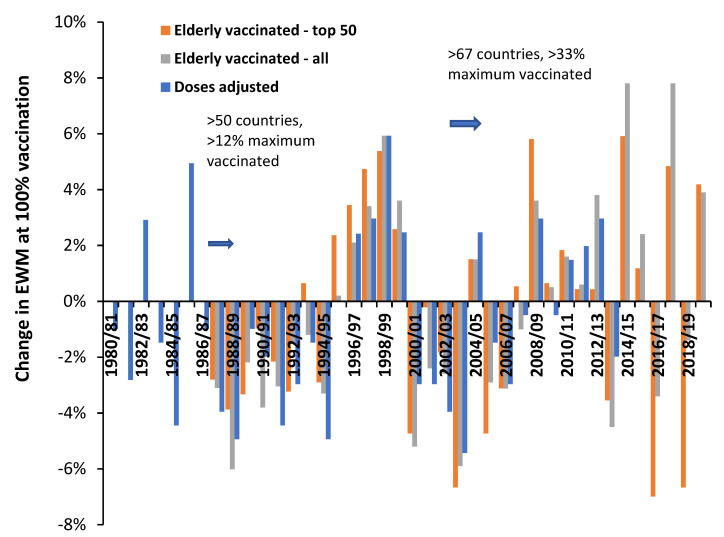
Comparison of the slope for the two large data sets.

**Figure 7:**
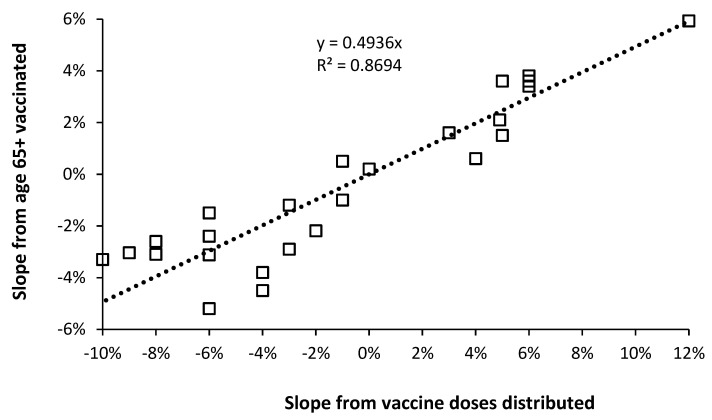
Comparison of calculated slope in the relationship between adjusted EWM and proportion vaccinated using either proportion elderly aged 65+ vaccinated or vaccine doses distributed per 1000 population, in the overlap years 1988/89 to 2012/13.

Does this data behave in the same way as that used in Figure 4? Figure 6 shows the output from such analysis where the slope of the EWM versus doses per 1000 population data is plotted alongside the slope for proportion elderly vaccinated data. The vaccine doses distributed data has first been adjusted for the fact that this method always gives a greater value than that from the elderly vaccinated data. This relationship is shown in Figure 7 where the slope from doses distributed must first be multiplied by 0.4936 to give an equivalent slope to that from the proportion aged 65+ study. From the comparison of the two data sets a further period of instability operates between 1980/81 and 1986/87. However, the point has been established that both data sets mirror each other.

### 4.5. The Values for the Slope Follow an Extreme Value Distribution

**Figure 8:**
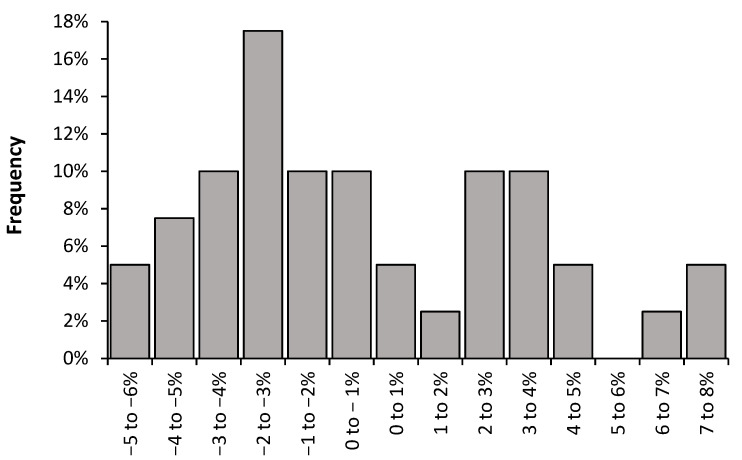
Frequency distribution for the calculated slope (as 1% increments) in the relationship between adjusted EWM and proportion vaccinated using either proportion elderly aged 65+ vaccinated or vaccine doses distributed per 1000 population, over the 40-year period 1980/81 to 2019/20.

Using the 40 years of available data, and ignoring the fact that the trend may have cyclic elements, allows analysis of the frequency distribution for the slope. The average value of the slope for each year was determined from #1, #2, #4 (plus 0.66% to account for the difference in the obesity adjusted slope identified in Section 4.2) above plus available data from the vaccine doses distributed data after adjustment as in Figure 7. Data was aggregated into 1% increments in the value of the average slope, and this is presented in Figure 8. The average value for the slope is −0.3%, the median value is −1.2% and a slope of −2% to −3% represents the most frequent value (the mode). The distribution is right skewed, and a negative slope occurs on 63% of occasions, while 58% of the values lie in the range 0% to −5%. The best description is that the shape of the distribution resembles an extreme value distribution, or possibly the outcome of two or more extreme value distributions. The implications of an extreme value distribution will be covered in Section 5.7 of the Discussion.

## 5. Discussion

This study does not in any way seek to claim that influenza vaccination does not offer a measure of protection against influenza induced death per se. We merely highlight that winter is a multi-pathogen complex system, and that unexpected or emergent outcomes should be expected as ‘normal’. Figure 1 and Figure 2 illustrate system complexity which seems far higher than could arise from the action and spread of a single pathogen, i.e., influenza.

### 5.1. What Is the “Real” Long-Term Effect?

Our earlier study suggested that higher rates of influenza vaccination appeared to make no effect on the long-term trend in EWM [26]. We proposed that this may be due to increasing (multi) morbidity in many countries acting to mask the effects of influenza vaccination. However, Figure 5 gives an alternative explanation in that the apparent slope of the relationship will depend on the time-period. The periods of benefit/disbenefit also help to explain the high variation associated with the proportion of age 65+ vaccinated in the earlier study. Recall that in the earlier study levels of vaccination increased over time.

Using the data behind Figure 4 and applying a 12-year rolling median/average (as an example of a randomly chosen period) the apparent median/average slope between 1996/97 to 2007/08 would be −1.5%/−0.5% respectively (net benefit), while the apparent slope between 2008/09 and 2019/20 would be +1.2%/+1.4% respectively (net disbenefit).

Hence, over the longer term, the years in which influenza vaccination has a net benefit is cancelled out by the years in which there is net dis-benefit. The rolling 12-year average in this study (using 12-years as a random example) goes from a net zero effect up to the 12-years ending 2008/09, reaches a maximum net *benefit* of −1.3% for the 12-years ending 2011/12 and then shows maximum net disbenefit of +1.4% for the 12-years ending 2019/20.

Hence the conclusion from this, and the previous study [26] that the “real” long-term slope is close to zero, is likely to be the best estimate, since the observed medium-term slope shows undulations over time. Clearly any effect due to the increasing proportion of persons aged 65+ vaccinated is being overwhelmed by other specific annual factors.

### 5.2. Limitations of Our Earlier Hypothesis

In our preceding paper we proposed that the benefits of increased influenza vaccination were being counterbalanced by rising levels of obesity and other (multi) morbidities [26]. The USA was used as a worst-case scenario. EWM in the USA was increasing at just 0.02% (percentage points) per annum [26]. A 0.8% (percentage point) increase in 40 years. However, the study of Simonsen et al. [28], which covered the somewhat shorter period of very rapid expansion in elderly influenza vaccination in the USA between 1987 to 1996 (a jump from 25% to 62% elderly vaccinated in just 9 years), was unable to detect any measurable effect on EWM.

Obesity and other (multi) morbidities only increase slowly over decades and would be totally unable to overwhelm the benefit of such a large and rapid expansion in elderly influenza vaccination. The same was observed to occur in Italy [29]. In Figure 6, 1987 to 1996 encompasses a 7-year period of moderate net benefit followed by a 4-year period of rapidly escalating net disbenefit (also illustrated in the CUSUM in Figure 5).

It is this switch from net benefit to net disbenefit which confounded the above-mentioned studies [28,39], rather than any small increment in obesity and (multi) morbidities. Indeed, as this study demonstrates, adjusting world countries to the equivalent US obesity level has little effect on the observed slope of the relationship each winter.

Hence, while we concede that increasing obesity and (multi) morbidities may act slowly over decades to erode the benefits of increasing elderly vaccination, it is likely that the more powerful annual effects far outweigh such long-term trends in human health status.

### 5.3. Adjustment for Obesity

The adjustment for obesity in Section 4.2 is an example of a single parameter model. As such, it is highly likely that obesity may be acting as a proxy for the wider morbidity issues discussed in the previous study [26], which are also increasing with time. The relatively low slope for the seeming effect of ‘obesity’, i.e., a 0.12% increase in EWM for each 1% increase in obesity seems to add weight to the proposal that rising levels of morbidities are not the cause of the apparent lack of effect of influenza vaccination observed over a 40-year period in the earlier study. The real reason lies in the annual effects reported in this study.

### 5.4. Implications of High International Variation

The high inter- and intra-national variation observed in Figure 1 and Figure 2, along with the high scatter around the trend line in Figure 3, leads to a low R-squared. An R-squared of 0.156 was quoted for the winter of 2017/18 (Figure 3) with a similarly low value for 2014/15 of 0.1336 (as a wider example). A low R-squared implies that the principal variable, i.e., proportion of persons age 65+ vaccinated, is only explaining 13% to 16% of the observed variation in EWM. This will partly be because influenza vaccine effectiveness (VE) is itself highly variable [27]. However, the low R-squared is probably more to do with the fact that winter is a multi-pathogen complex system. Hence influenza vaccination per se is unable to exert much control over the variation in EWM. This concurs with the sometimes-unexpected results presented in Figure 4 and Figure 6.

### 5.5. 2014/15 as an Example of Poor Vaccine Matching

As can be seen in Figure 4 and Figure 6 there are only 2 years with a very high net protective effect from influenza vaccination (1988/89 and 2000/01), but four years with a very high net disbenefit (1998/99, 1999/2000, 2014/15, 2017/18) and all the net disbenefit years correspond with very high EWM. Hence it is difficult to claim that influenza alone was responsible for the higher deaths in these four high years.

The winter of 2014/15 can be used as an example since in Figure 1 it is characterized as having the highest upper quartile for world countries, as also seen in Figure 2 for UK LGAs. The Public Health England summary report covering the whole UK noted that levels of influenza-like-illness (ILI) were barely above baseline [53], which was entirely insufficient to explain the unusually high mortality. Influenza A predominated early in late 2014 to early 2015 while B predominated after week 10 in 2015. Most excess deaths occurred in 2015 [53]. However, a series of antigen mismatches in both influenza(s) A and B between the vaccine used that year and the strains and variants which circulated were noted [53]. The report also noted that “A portion of 2014 to 2015 influenza A(H3N2) viruses did not grow sufficiently for antigenic characterization” [53]. Hence, additional hidden antigenic complexity may be involved.

Vaccine uptake for those aged 65+ across the UK ranged from 68% to 76% for the four countries in the union. A mid-season estimate of vaccine effectiveness (VE) for influenza A was only 2.3% (range −48.5% to +36.1%). Influenza A(H3N2) had a VE of only 0.6%. No VE for influenza B was given in this report [53]. VE in Canada and several other countries went negative, and unusual patterns of small area deaths were noted across England and Wales [54]. Respiratory syncytial virus (RSV) was also active [53].

It should be noted that VE in the UK is determined on (ambulatory) General practitioner (GP) visits and hence may overestimate VE relating to deaths. For example, in a Swiss study persons aged 65+ admitted to hospital with ILI were 7.5-times more prevalent than the ILI visits to a GP surgery and the GP sample contained 5.8-times more aged 5–14, and 4.5-times more aged 15–29 [55]. Presentation at the hospital also commenced earlier than in the community [55].

Influenza activity and excess deaths during the earlier 2014 winter in Australia (southern hemisphere) were unremarkable [56], hence, the emerging strains/variants which contributed to high EWM in the northern hemisphere likely became more prevalent after September of 2014. It is unknown when and where they originated.

We propose that low and possibly negative VE (for death) is seemingly associated with the unusually high winter deaths seen in both the UK and other northern hemisphere world countries during 2014/15. This has partly contributed to the observed net disbenefit due to influenza vaccination that year.

### 5.6. Roles for Pathogen Interference

Winter is a multi-pathogen event [22,57,58,59], and multiple pathogens cause influenza-like-illness (ILI), and death [59]. Interaction between pathogens is very common and is termed ‘pathogen interference’ [60,61]. Pathogen interference in coinfections can diminish or augment infection by other pathogens and has direct clinical consequences [62].

Since pathogen interference is not a widely appreciated phenomenon, it has been claimed that the imposition of lockdowns during the COVID-19 pandemic were responsible for the early decline in influenza activity during the winter of 2019/20 [63,64]. However, close inspection of weekly influenza activity figures in the UK show very clearly that influenza activity had dropped to baseline levels by week 3 of 2020 and had declined to zero during week 12 [65]. Lockdown in the UK legally came into force on Thursday 26th March 2020 [66] which is just at the point when influenza activity had already dropped to zero. In the UK, lockdowns cannot in any way be said to have contributed to the fall in influenza activity which commenced its rapid decline much earlier in the year when COVID-19 spread was gaining momentum [65].

In Canada during the 20-week period after week 11 of 2020 compared to the pervious 148 weeks a 70% decline in influenza prevalence was observed. However, respiratory syncytial virus (RSV) only declined by 54%, parainfluenza virus (PIV) declined by 60%, but coronaviruses (hCoVs) (excluding COVID-19s) increased by 80%, metapneumoviruses (HMPV) increased by +45%, and entero/rhino viruses (hERV) by +40% [64]. These results indicate that while protective measures may have played a limited role, additional virus-specific factors were specifically involved.

Of even greater relevance to pathogen interference is the virtual extinction of influenza B/Yamagata during the COVID-19 pandemic [67]. In Israel, pneumococcal disease in young children radically reduced during the first year of COVID-19, mainly due to suppression of RSV, influenza viruses, and hMPV. However, hERV and PIV activities were within or above expected levels [68].

In the USA influenza and RSV activity were initially suppressed by COVID-19, However, RSV then underwent an unusual resurgence during the summer of 2021 [69]. This study also demonstrated that there was considerable variation in the reduction in influenza activity between US states, with a 79% reduction in Texas through to a 28% increase in Idaho [69]. Coinfection with influenza and COVID-19 occurs at low frequency, although coinfection appears to occur more often in Asia than the USA [70].

To explain all the above requires that pathogen interference between COVID-19 and other viruses is the predominant explanatory force. We propose that pathogen interference, which has been active for many centuries, has a major role in the observed long-term cycles in EWM detailed in the previous study [26] and during the COVID-19 era.

### 5.7. Could Vaccine Effectiveness Be an Illusion Created by Pathogen Interference?

The introduction of PCR-confirmed ‘test negative’ influenza VE commenced around the early 2000s and is well recognized to rely on the assumption that the levels (and pathogenicity) of non-influenza pathogens is identical in both groups [71]. However, earlier studies consistently reported lower *net* VE. For example, the study of Fireman et al. [72] found that influenza vaccination only reduced mortality by 4.6% over 9 flu seasons. Note that the design of this study is such that this is a net reduction, i.e., the net effect in a multi-pathogen complex system.

Several studies do exist which suggest that pathogen interference is active after influenza vaccination in children [73,74], during pregnancy [75] and in the elderly [76]. Such observations question the fundamental assumptions behind the calculation of VE and indicate a shift to higher infection by non-influenza pathogens. As an aside, pregnancy is an example of a temporary immune steady state [77]. Indeed, the immune response to influenza vaccination is recognized to exhibit variation between individuals [78] and has been proposed to alter the balance of pathogen interference and affect the optimum timing of vaccination [79]. In light of the findings in this study this area requires far greater investigation.

### 5.8. Heliobiology and Additional Hidden Complexity

As can be seen in Figure 4, Figure 5 and Figure 6 the data seems to become more volatile/unstable during two periods from 1980/81 to 1986/87 and 2000/01 onwards. We propose a potential relationship with fluctuations in solar radiation or, more correctly, coronal mass ejections (CME) [80].

Solar output of electromagnetic radiation (7% X-ray, gamma-ray and ultraviolet, 44% visible, 49% microwave, infrared and radio wave) is surprisingly volatile even at the level of seconds and minutes [81]. These fluctuating emissions are due to coronal mass ejections (CMEs), which also include high energy protons, and tend to occur more often (but not always) at periods when solar flares are most active [80]. One of the observable effects of these solar storms (CMEs) are electrical power grid anomalies (power surges and electrical transformer failures) which arise from geomagnetically induced currents [82].

CMEs and resulting electromagnetic levels have been linked to short-term fluctuations in human health, immune function, morbidity, and mortality called heliobiology [83]. One review concluded that 10–15% of the population are predisposed to the adverse effects of geomagnetic variations [84]. Patients with multiple sclerosis show enhanced hospital admission during periods of geomagnetic disturbance [85]. Obscure phenomena, such as sudden infant deaths, appear to rise with sunspot activity (by implication CMEs) [86]. Geomagnetic field fluctuations have been observed to alter gene expression [87]. Several studies have suggested that influenza pandemics are aligned with the solar cycle [88,89]. COVID-19 and other pathogen outbreaks all appear to fall into the same pattern [90]. We propose that CMEs add a hidden layer to the already complex behavior observed in the previous study [26] and this study.

We offer the following tentative observations; namely, the points at which the CUSUM in Figure 5 changes to a positive slope around 1994/95 and 2007/08 occur as sunspot cycles 22 and 23 approach their minima. The two periods of instability both correspond to very intense instances of sunspots at the peaks of sunspot cycles 22 and 24. The CUSUM slope goes negative after the intense parts of the peaks in sunspot cycles 22 and 23, see chart in reference [91]. We stress this is tentative evidence, since CME magnitude and timing does not exactly follow sunspot cycles. However, a body of evidence appears to be accumulating.

### 5.9. Implications of an Extreme Value Distribution for the Slope

Figure 8 demonstrated that the slope of the annual relationship appeared to be an example of an extreme value distribution. Extreme value distribution is commonly used to describe natural events such as temperature variation, rainfall, river flow, flooding, and stock market volatility [92]. The implication is that the volatility in the slope is subject to natural world complexity in which the minimum value of the slope, i.e., influenza vaccination is net protective, i.e., has a lower boundary, while the upper boundary can exhibit extreme values, i.e., influenza vaccination promotes net disbenefit. Roles for CMEs (Section 5.6) and other potential contributory factors need to be further explored. It is fundamentally important to understand which factors trigger the unexpected adverse net effects of influenza vaccination. In practice, CMEs are very difficult to quantify (apart from directly measuring the electromagnetic flux at different points on the Earth’s surface) and are highly likely to show extreme spatiotemporal variation.

### 5.10. Minimum Value of the Slope

In the previous study, a minimum possible slope of −10% was assumed [26] and this corresponds to −6% at 60% VE. A VE of 60% is the highest VE for persons aged 65+ ever reported in the USA [27]. A slope of −6% is demonstrated in this study to only occur once in 40 years. This study therefore questions the preliminary suggestion made in the earlier study that obesity and other (multi) morbidities may be masking the effects of influenza vaccination [26]. This is especially relevant in that adjustment of annual data for the effects of obesity in Figure 4 made little effect on the slope of the relationship (also discussed in Section 4.5). Recall that in Figure 4 the effect of obesity is most likely to be serving as a proxy for wider time-related changes in multiple morbidities [26].

### 5.11. Biochemical and Immune Health

One study has implicated roles for biochemical health in the response to vaccination [46]. A large study is relevant to this concept. In this study the results from common biochemical tests were combined into a composite score [92]. The interesting observation was that humans had a wide range for the composite score, which was, however, relatively stable over time for each person. The population average for this score (biochemical health) only showed a small decline with age but showed a rapid decline in the weeks and months preceding death [93]. This is consistent with the nearness-to-death effect [94], where frailty, cognitive function, perceived physical health and mental wellbeing, etc., only show a rapid change as death approaches [95,96,97,98]. The suspicion is that nearness-to-death, howsoever determined, is a completely neglected variable and may imply that birth cohort effects play an additional role in long-term trends and vaccine effectiveness [99,100,101]. This point is raised in the context of additional hidden system complexity.

The immune system consists of specialized cell populations that communicate with each other to achieve systemic immune responses. Analysis of various immune cell population frequencies in healthy humans and their responses to diverse stimuli showed that human immune variation is continuous in nature, rather than characterized by discrete groups of similar individuals (as observed for the composite biochemical score study above) [102]. Three combinations of immune cell population frequencies were observed to define an individual’s immunotype and predict a set of functional responses to cytokine stimulation. Even though inter-individual variations in specific cell population frequencies can be large, unrelated individuals of younger age had more homogeneous immunotypes than older individuals. Across age groups, cytomegalovirus seropositive individuals displayed immunotypes characteristic of older individuals. The conceptual framework for defining immunotypes suggests the development of better therapies that appropriately modulate collective immunotypes, rather than individual immune components [102].

The above suggests that certain individuals may be more susceptible to the unintended adverse effects of influenza vaccination. This possibility requires further investigation. Indeed, do persons in the terminal decline phase of life, which occurs in the last year of life, benefit equally from influenza vaccination? There are gaps in our understanding, which may be relevant to the unintended net effects of influenza vaccination.

### 5.12. A Potential Basis for Extreme Variation in the Net Effects of Influenza Vaccination

The thrust of this paper has been that well intended interventions into a highly complex system are likely to generate unexpected outcomes. This is supported by wider research in complexity theory [30,31,32,33]. We have highlighted instances of immune and biochemical health, and of heliobiology, where differences exist among individuals within a population. We would also like to point out that the immune manipulating persistent virus, cytomegalovirus, has a major reservoir of infection in the lung [103]. This virus has been proposed to interact with influenza in the lung; however, CMV, likewise, seems to affect some individuals more so than others. All of this is then within the context of pathogen interference and the potential unintended effects of influenza vaccination upon pathogen balance and the immune response of different people.

## 6. Pragmatic Implications to Health Care Services

Influenza vaccination is widely recommended by public health agencies as a route to reducing health service winter pressures. One of the contributing factors to this study was the observation that increasing influenza vaccination rates did not seem to be making a net contribution to the reduction in hospital winter capacity pressures [104]. This seemed to contradict the known ability of influenza vaccination to reduce influenza-related hospital admissions and death. This study confirms this earlier observation that deaths, and the associated acute care prior to death, are showing unexpected outcomes.

## 7. Implications to Influenza Policy

The economic rationale for influenza vaccination partly relies on the assumption that it has a net beneficial effect against deaths [105]. The implications of the earlier study [26] and this study question this assumption. Furthermore, two large regression discontinuity studies have demonstrated that at the age 65 boundary, where influenza vaccination is widely recommended, there is no statistically detectable net benefit against hospital admission and deaths [106,107]. The possibility exists that estimating influenza VE for the age 65+ group—an age range which is far too wide—is concealing further complexity, in that age is acting as a poor proxy for nearness-to-death. Policy must be based on facts and not upon flawed single pathogen, simple system behavior assumptions.

## 8. Limitations and Future Research

This study is limited by the availability of monthly data. The majority of Africa has no data and data from Asia and South America is limited. Countries with larger states/provinces/regions should confirm the results of this study using sub-national data. Brazil is an ideal example, since it spans the equator. Total proportion vaccinated (all-age) or age 65+ vaccinated can be used depending on data availability. Potential roles for other winter pathogens need to be clarified. The adjustment factor based on median EWM may need to be refined given the long-term cycles which seemingly characterize the trends in EWM [26]. It is unknown if these cycles are country-specific or are driven by other factors, such as heliobiology.

The exact role of obesity, other morbidities and polypharmacy remains to be accurately quantified—although they represent long-term trends. Regarding the risk of death due to COVID-19, it has been noted that “polypharmacy may represent a marker of vulnerability, especially for younger groups of older adults” [108]. Japan, South Korea, and Singapore can serve as low obesity benchmarks. Future studies on this topic could use weighted regression without trimming; however, this is unlikely to make a material change to the conclusions.

However, even after assuming a moderately high contribution for obesity upon EWM no significant effect could be demonstrated on the slope of the annual data.

## 9. Conclusions

This study has demonstrated that unexpected or emergent behavior is indeed occurring as an unintended effect of widespread influenza vaccination. Adverse outcomes regarding net winter mortality after influenza vaccination occur in roughly 40% of years. However, the exact relationship appears to follow long-term cycles. The existence of such cycles was demonstrated in a previous study [26]. This study appears to confirm the predictions made in the 2010 study of Berencsi et al. [79] that vaccination has the potential to alter pathogen balance. One of the points made in their study was that the timing of vaccination may need to be modified to account for time-based prevalence of other pathogens [78]. This is a testable hypothesis, given that the date of vaccination for individuals is generally readily available and that many countries also have data on the prevalence of other common winter pathogens.

## Figures and Tables

**Table 1:** Searches on influenza system complexity using Google Scholar. Search conducted on 6th October 2021.

Search String	Documents Identified
Influenza epidemics “complex systems”	94,800
Influenza and “systems biology”	22,000
Complex system dynamics pandemic influenza	18,800
Interactions influenza and “other pathogens”	16,200
Influenza and “pathogen interactions”	14,600
Influenza and “complex system”	10,900
Influenza vaccination and “complex system”	4520
Influenza and “pertussis complex relationship”	570

## Data Availability

All data is publicly available. Copies of various data tables can be obtained from Rodney Jones on request, email: hcaf_rod@yahoo.co.uk.

## Supplementary Materials

The following supporting information can be downloaded at: https://www.mdpi.com/article/10.3390/idr14030035/s1, Table S1: Relationship between the obesity factor and the resulting correlation between All Country slope and Obesity adjusted slope. Table S2: Summary of data used in the study including influenza vaccine doses distributed and proportion aged 65+ vaccinated versus adjusted EWM.
