# Supplementary material for: System Complexity in Influenza Infection and Vaccination: Effects upon Excess Winter Mortality
Source: Infect Dis Rep. 2022 Apr 21;14(3):287–309. doi: 10.3390/idr14030035 (PMC9149983; doi:10.3390/idr14030035)
Supplement: Supplementary file 1 [file idr-14-00035-s001.zip › idr-1674367-supplementary.pdf]

infectious disease reports

MDPI

Supplementary material

Table S1. Relationship between the obesity factor and the resulting correlation between All Country slope and Obesity adjusted slope

Obesity Factor	R-squared	Slope
0.30	0.9622	0.9449
0.20	0.9791	0.9751
0.15	0.9798	0.9815
**0.12**	**0.9799**	**0.9855**
0.10	0.9798	0.9880
0.07	0.9793	0.9919
0.05	0.9789	0.9945
0.02	0.9780	0.9984

Table S2 next page …….

Table S2. Summary of data used in the study including influenza vaccine doses distributed and proportion aged 65+ vaccinated versus adjusted EWM. Count = number of countries with available data. The slope from vaccine doses distributed is raw and adjusted using the relationship in Figure 7. The slope for the "Obesity adjusted" data uses an assumed 0.12% increase in EWM per 1% increase in adult obesity. This slope is then adjusted down by 0.37% to account for the change in intercept arising from the obesity adjustment. The "All variance" column gives the variance associated with the "All countries" data using the age 65+ vaccinated data. STDEV = standard deviation of the average slope. Before 1987/88 the maximum age 65+ vaccinated was not available. The low median for age 65+ vaccinated prior to 1996/97 is due to increasing numbers of countries with zero or low vaccination rates. The average value of the slope and STDEV are calculated from the adjusted slope from doses distributed plus age 65+ data covering All, +US states, and Obesity adjusted. The STDEV of the average slope tends to decline as the slope gets higher, -0.0377 for every percent increase in the slope. However, the R-squared is only 0.183 indicating that average slope only explains 18.3% of the variance in STDEV.

Year	Age 65+ vaccinated		Count			Intercept				Slope (doses distributed)		Slope (age 65+ vaccinated)				All Variance	Slope	
	Median	Maximum	Doses Distributed	Age 65+ All	Age 65+ Top 50	All	Top 50	+ US states	Obesity adjusted	Raw	Adjusted	All	Top 50	+ US states	Obesity adjusted		Average	STDEV
1980/81			34							-2%	-1.0%						-1.0%	n/a
1981/82			41							-6%	-2.8%						-2.8%	n/a
1982/83			40							6%	2.9%						2.9%	n/a
1983/84			41							-3%	-1.5%						-1.5%	n/a
1984/85			35							-9%	-4.4%						-4.4%	n/a
1985/86			42							10%	4.9%						4.9%	n/a
1986/87			39							-2%	-1.0%						-1.0%	n/a
1987/88	3%	44%	41	50	37	11.1%	11.2%		12.2%	-8%	-3.9%	-4.3%	-4.7%		-4.9%	0.09%	-4.4%	0.5%
1988/89	4%	45%	41	51	39	11.2%	10.6%		12.1%	-10%	-4.9%	-6.0%	-3.6%		-6.0%	0.09%	-5.6%	0.6%
1989/90	1%	49%	53	53	40	13.7%	14.0%		15.0%	-2%	-1.0%	-2.2%	-3.1%		-3.1%	0.09%	-2.1%	1.1%
1990/91	1%	58%	54	59	43	12.0%	11.0%		12.9%	-4%	-2.0%	-3.8%	-0.9%		-3.7%	0.09%	-3.1%	1.0%
1991/92	2%	59%	57	60	48	12.0%	11.7%		13.3%	-9%	-4.4%	-3.0%	-2.0%		-3.5%	0.06%	-3.7%	0.7%
1992/93	4%	59%	56	58	46	13.1%	13.6%		14.4%	-6%	-3.0%	-1.5%	-3.0%		-3.7%	0.09%	-2.7%	1.1%
1993/94	5%	59%	60	61	49	13.2%	12.6%		14.8%	-3%	-1.5%	-1.2%	0.6%		-2.5%	0.12%	-1.7%	0.7%
1994/95	6%	62%	58	60	46	11.7%	11.3%		13.2%	-10%	-4.9%	-3.3%	-2.7%		-4.1%	0.11%	-4.1%	0.8%
1995/96	6%	66%	47	53	40	13.5%	12.6%		15.3%	0%	0.0%	0.2%	2.2%		-1.0%	0.15%	-0.3%	0.7%
1996/97	7%	68%	52	62	47	13.4%	13.0%		15.1%	5%	2.4%	2.1%	3.2%		1.4%	0.08%	2.0%	0.5%

Year	Age 65+ vaccinated		Count			Intercept				Slope (doses distributed)		Slope (age 65+ vaccinated)				All Variance	Slope	
	Median	Maximum	Doses Distributed	Age 65+ All	Age 65+ Top 50	All	Top 50	+ US states	Obesity adjusted	Raw	Adjusted	All	Top 50	+ US states	Obesity adjusted		Average	STDEV
1997/98	8%	70%	52	57	46	10.8%	10.2%		12.6%	6%	3.0%	3.4%	4.4%		2.6%	0.13%	3.0%	0.4%
1998/99	9%	70%	48	58	47	13.0%	13.5%		14.7%	12%	5.9%	5.9%	5.0%		5.7%	0.17%	5.9%	0.1%
1999/00	11%	76%	59	66	47	15.0%	15.5%		17.1%	5%	2.5%	3.6%	2.4%		2.6%	0.14%	2.9%	0.6%
2000/01	17%	76%	59	68	47	11.6%	11.3%		13.4%	-6%	-3.0%	-5.2%	-4.4%		-5.2%	0.11%	-4.4%	1.3%
2001/02	18%	78%	63	73	46	11.8%	10.6%		14.2%	-6%	-3.0%	-2.4%	-0.2%		-3.6%	0.11%	-3.0%	0.6%
2002/03	21%	78%	67	74	49	13.1%	12.8%		15.0%	-8%	-3.9%	-2.6%	-1.7%		-2.7%	0.15%	-3.1%	0.7%
2003/04	20%	77%	64	76	46	13.9%	13.8%		16.2%	-11%	-5.4%	-5.9%	-6.2%		-6.7%	0.12%	-6.0%	0.6%
2004/05	24%	82%	67	74	46	14.4%	14.6%		16.7%	5%	2.5%	1.5%	1.4%		-0.2%	0.10%	1.2%	1.4%
2005/06	25%	84%	69	79	48	11.9%	12.7%		14.1%	-3%	-1.5%	-2.9%	-4.4%		-3.4%	0.13%	-2.6%	1.0%
2006/07	24%	82%	70	83	50	12.1%	11.6%		14.4%	-6%	-3.0%	-3.1%	-2.9%		-3.7%	0.11%	-3.3%	0.4%
2007/08	25%	81%	73	80	48	12.0%	11.3%	11.4%	13.9%	-1%	-0.5%	-0.4%	0.9%	-0.1%	-0.4%	0.11%	-0.3%	0.2%
2008/09	30%	83%	69	76	48	11.0%	10.4%	11.6%	13.1%	6%	3.0%	4.0%	5.4%	3.3%	4.0%	0.13%	3.6%	0.5%
2009/10	27%	88%	71	73	45	10.6%	10.7%	10.7%	13.1%	-1%	-0.5%	0.3%	0.1%	-0.1%	-0.3%	0.06%	-0.2%	0.3%
2010/11	25%	81%	69	77	48	9.5%	8.6%	9.1%	11.9%	3%	1.5%	2.6%	3.6%	2.7%	2.3%	0.07%	2.3%	0.6%
2011/12	25%	80%	71	80	49	12.4%	12.5%	13.4%	15.1%	4%	2.0%	0.6%	0.4%	-0.6%	0.1%	0.17%	0.5%	1.1%
2012/13	26%	80%	71	84	50	10.9%	13.0%	10.3%	14.1%	6%	3.0%	3.8%	0.4%	4.5%	2.3%	0.19%	3.4%	1.0%
2013/14	25%	81%	74	85	50	12.6%	11.2%	12.1%	15.3%	-4%	-2.0%	-4.5%	-3.3%	-3.8%	-5.3%	0.16%	-3.9%	1.4%
2014/15	25%	84%		85	49	13.0%	14.7%	13.4%	15.7%			7.8%	5.5%	7.7%	7.0%	0.23%	7.5%	0.5%
2015/16	26%	84%		81	45	9.8%	10.2%	10.4%	12.7%			2.4%	1.1%	1.8%	1.5%	0.11%	1.9%	0.5%
2016/17	27%	85%		79	45	16.7%	18.6%	16.6%	19.8%			-3.4%	-6.5%	-3.5%	-4.6%	0.22%	-3.8%	0.7%
2017/18	24%	97%		80	45	11.9%	14.8%	12.6%	14.7%			7.8%	4.5%	7.2%	7.2%	0.19%	7.4%	0.4%
2018/19	30%	97%		75	46	12.7%	15.0%	13.0%	16.1%			-1.4%	-6.2%	-1.4%	-2.8%	0.11%	-1.8%	0.8%
2019/20	36%	86%		66	45	9.6%	9.2%	10.0%	9.9%			3.9%	4.5%	3.9%	3.2%	0.11%	3.7%	0.4%
